# Herbal Additives Substantially Modify Antioxidant Properties and Tocopherol Content of Cold-Pressed Oils

**DOI:** 10.3390/antiox10050781

**Published:** 2021-05-14

**Authors:** Kamila Laskoś, Elżbieta Pisulewska, Piotr Waligórski, Franciszek Janowiak, Anna Janeczko, Iwona Sadura, Szymon Polaszczyk, Ilona Mieczysława Czyczyło-Mysza

**Affiliations:** 1 The *Franciszek Górski Institute* of Plant Physiology, Polish Academy of Sciences, Niezapominajek 21, 30239 Kraków, Poland; k.kaploniak@ifr-pan.edu.pl (K.L.); p.waligorski@ifr-pan.edu.pl (P.W.); f.janowiak@ifr-pan.edu.pl (F.J.); a.janeczko@ifr-pan.edu.pl (A.J.); i.sadura@ifr-pan.edu.pl (I.S.); 2 Carpathian State College in Krosno, Rynek 1, 38400 Krosno, Poland; elzbieta.pisulewska@gmail.com; 3 Andrzej Frycz Modrzewski Krakow University, Gustawa Herlinga-Grudzińskiego 1, 30705 Kraków, Poland; szympol@poczta.fm

**Keywords:** oily herbal plants, macerates, *Salvia officinalis* L., *Thymus vulgaris* L., *Ocimum basilicum* L., oil cake, tocopherols, antioxidants activity

## Abstract

The aim of the study was to examine combinations of base oils and herbal additives with a view to obtaining macerates with improved health benefits. Base oils were cold-pressed from the seeds of black cumin, borage, evening primrose, safflower, walnut, common hazel, and oilseed rape, as well as the flesh of sea-buckthorn fruits. They were then supplemented with herbs, including basil, thyme, and sage, in order to create macerates. Total antioxidant activity and tocopherol level were analyzed in oils, macerates, and oil cakes. Additionally, chemical properties of oil cakes—such as the level of fibre, vitamin C, β-carotene, and lutein—were also examined. Supplementation with herbs caused diversified effects on antioxidant activity and tocopherol level in macerates depending on the base oil, herb, and supplementation method. The obtained results indicate that tocopherol level does not play a decisive role in determining the antioxidant properties of oils, macerates, and oil cakes, suggesting significant involvement of other antioxidants. Among the tested macerates, the most promising one seems to be oilseed rape oil enriched with sage or basil to maximize its health benefits. The study can serve as a starting point for the development and implementation of functional macerates and oil cakes in healthy nutrition.

## 1. Introduction

Cold-pressed fats and oils are edible products obtained by mechanical procedures, such as expelling or pressing, during which no heat is applied, and no modification of the oil occurs. Purification of these oils can only occur through water washing, settling, filtration and centrifugation [1]. Cold-pressing preserves a large amount of antioxidants (e.g., tocopherols, polyphenolic compounds) and also polyunsaturated fatty acids of the n-3 and n-6 group as well as sterols, indicating a potential of cold-pressed oils in antioxidant and bioactive effects [2,3,4,5]. In contrast to cold-pressing, the refining process causes a partial removal of nutritionally valuable compounds such as tocopherols, carotenoids, sterols, and polyphenolic compounds [6,7,8,9,10,11]. Therefore, there is a noticeable interest in cold-pressed oils among consumers looking for food with healthful properties.

Various species contain different amounts of antioxidants. Antioxidants are an important component in human nutrition, protecting the body’s molecules against oxidation and, as a consequence, destruction. Thus, dietary antioxidants help protect the human body against oxidative stress. 

Living conditions in industrialized countries are especially conducive to the development of oxidative stress, and the multitude of stimulating factors only exacerbates this phenomenon. Therefore, a diet rich in bioactive compounds, especially antioxidants, is currently one of the solutions that can help protect organisms against the destructive effects of oxidative stress [2,12]. Other mechanisms activated by plant extracts rich in antioxidants have also been demonstrated in the literature, such as immunomodulating activity [13,14], or anti-proliferative and pro-apoptotic effects on cancer cells [15,16]. Furthermore, studies are being carried out into the effectiveness of plant extracts in reducing cellular senescence of human cells. Yang [17] suggests that they represent a promising possibility to block age-related loss of tissue functionality.

Moreover, plant extracts could be used to develop dietary supplements or cosmetics for modulating tissue aging and aging-associated diseases [17,18]. Oily herbal plants are a group of plants characterized by the presence of essential unsaturated fatty acids (EFA), such as linoleic acid (n-6 octadecadienoic acid), α-linolenic acid (n-3 octadecatriene acid), and compounds belonging to their families [arachidonic acid (eicosatetraenoic acid, n-6), eicosapentaene, n-3 (EPA) and docosahexaene, n-3 (DHA)]. Some of the species, especially those rich in EFAs, are used in medicine, dietetics and cosmetology [19]. They include the species used in this study—black cumin (*Nigella sativa* L.), safflower (*Carthamus tinctorius* L.), borage (*Borago officinalis* L.) and evening primrose (*Oenothera biennis* L.). Herbal oils, obtained from oily herbal plants are also a source of antioxidants, fat-soluble vitamins (A, D, E), carotenoids and phytosterols [20,21,22,23,24,25,26]. Pressed at low temperatures and ingested raw, these oils are used, among others, in the prevention and treatment of cardiovascular diseases, atherosclerosis and cancerous diseases [19,27,28,29,30,31,32]. They are also used in dermatology (atopic skin, psoriasis), and as a component of nutricosmetics and cosmetics used in anti-aging skincare, UV ray protection and hair conditioning [33,34,35,36].

Despite their health benefits, the use of herbal oils in daily nutrition remains relatively rare and regional. The reason is both the short shelf life and the higher price compared to other vegetable oils. Vegetable fats rich in EFAs are easily oxidized (rancid) [37,38], and their durability is determined by the content of vitamin E (tocopherols and tocotrienols). A treatment that increases the stability (durability) of the vegetable oils is the addition of plant extracts rich in antioxidants. This role is best fulfilled by oily herbal plants and herbs which, apart from essential oils, contain a number of other substances with strong antioxidant properties, including glycosides–anthocyanins, flavonoids, carotenoids, phenolic acids, and also volatile compounds, catechins and vitamins (C, A, E) [39,40,41,42,43,44].

Herbal macerates are primarily used in cosmetics so far, and the oils used for their preparation are popular edible vegetable oils, most often rapeseed and sunflower oil. It is worth mentioning that before adding the herbal material, the oil is usually heated [45,46], which may result in the loss of valuable properties. Moreover, specialist literature lacks detailed information on the selection of base oils for particular herbal plant species, and above all, there is no analysis of the fatty acid composition and antioxidant content in macerates as compared to the raw materials.

The residue obtained after pressing the oils is the oil cake. Depending on the raw material, it is characterized by different composition and antioxidant activity [47]. Under mass production conditions, it is most often used as feed for ruminants, and in the case of oil pressing on a small scale or at home, they are a valuable dietary supplement rich in dietary fibre and antioxidants [48,49].

The aim of the study was to examine combinations of base oils and herbal additives with a view to obtaining macerates with improved pro-health properties compared to pure base oils. For this purpose total low molecular weight antioxidant activity and tocopherol level were measured in oils cold-pressed from the plants of eight species (oilseed rape, black cumin, safflower, hazel, borage, walnut, evening primrose, sea-buckthorn), and macerates obtained through the addition of herbs from the *Lamiaceae* family— common basil, sage, and thyme. Additionally, the chemical properties of oil cakes left after the cold-press of oil were analyzed by measuring the level of fibre, vitamin C, β-carotene, lutein, total tocopherols and total antioxidant activity.

## 2. Materials and Methods

### 2.1. Materials and Experiment Design

The study was conducted on base oils, macerates (base oils + herbs) and oil cakes obtained after pressing base oils from raw materials (Table 1). Base oils, constituting reference samples (control oil), were prepared from the following raw materials: seeds of black cumin (*Nigella sativa* L.), borage (*Borago officinalis* L.), evening primrose (*Oenothera biennis* L.), safflower (*Carthamus tinctorius* L.), walnut (*Juglans regia* L.), common hazel (*Corylus avellana* L.), and oilseed rape (*Brassica napus* L.), as well as the flesh of sea-buckthorn berries (*Hippophae rhamnoides* L.) (Table 1). Seeds and berries were bought at certified suppliers, providing certificates of origin and variety. After visual qualitative evaluation, raw materials were pressed on a slow-speed screw press (Farmet, UNO 1,1 kW, Czech Republic), which made it possible to control the pressing temperature. All the oils were pressed in the temperature range of 33–35 °C, and immediately afterwards gravity filtered and allowed to cool to a temperature of about 15 °C, then poured into tightly closed containers. The sea-buckthorn berries were first macerated with rapeseed oil in a 1:1 proportion, and then the oil was obtained using the same procedure as in the case of seeds.

Oil cakes, remaining as the residue from the oil pressing process, were packed in paper packages immediately after pressing, cooled to a temperature of 8–10 °C, and stored in a refrigerated counter.

In addition to the base oils described above, the following plants were used to produce macerates: sage (*Salvia officinalis* L.), thyme (*Thymus vulgaris* L.), and basil (*Ocimum basilicum* L.). Macerates were prepared separately for each herb. Any raw material with morphological changes or pathogen damages was eliminated. After the initial selection, herbal material was divided into portions in the forms and weights as shown in Table 1. 

Herbal material, prepared according to the above-mentioned scheme (Table 1), was transferred to washed and scalded glassware. Then, base oil in the amount of 250 mL was poured over the herbal material. The tightly closed vessels were placed in a dark room at a constant temperature of 15 °C for a period of 10 days. After this period, the macerates were poured into tightly closed 250 mL dark glass containers (bottles). The collected material was stored in a refrigerated counter at 8–10 °C, and subsequently subjected to further biochemical analyses. The total number of studied combinations was 160, i.e., 8 oil cakes and 152 oils (8 base oils and 144 macerates).

### 2.2. Biochemical Analysis

The analyses of the content of tocopherols (α, γ and δ tocopherols) and total low molecular weight antioxidant activity were carried out in the base oils, their oil cakes and the obtained macerates. Additionally, analyses of fibre, vitamin C, β-carotene and lutein were performed for the oil cakes.

#### 2.2.1. Tocopherols, β-Carotene and Lutein

Two types of samples were analyzed—oils and oil cakes. Chemical analyses of oils were conducted directly on oil samples, whereas oil cakes were mixed with chloroform, homogenized mechanically in a mortar, sonicated in ultrasonic bath (Polsonic Sonic 5, Warszawa, Poland) for 10 min, then incubated at 4 °C for 24 h and finally centrifuged (3500× *g* for 10 min). The supernatant was collected and analyzed with the same method as raw oil. For each combination—one type of base oil with additives or one type of oil cake—9 measurements were performed, 3 measurements on 3 independent samples, giving a total of 456 oil samples + 24 oil cakes samples = 480 samples and standards of the tested compounds.

Tocopherols (in base oil and oil cakes), β-carotene and lutein (in oil cakes) were analysed with HPLC-MS method based on modified protocol of Zhang et al. [50], where saponification step was eliminated. Briefly, 100 µL samples (oil or chloroformic extract) were dissolved in 0.5 mL chloroform and 0.5 mL phase A (described below). These samples were injected to Agilent Technologies 1260 HPLC coupled to 6410 Tandem Mass Spectrometer. The apparatus was equipped with Waters NOVA-PAK C18 4 μm 3.9 × 300 mm HPLC column, phase A was acetonitryle/methanol/water (72/8/3 *v/v/v*) mixture and phase B was methanol/ethyl acetate (68/32 *v/v*) mixture. Flow rate was 1 mL/min, temperature was set to 35 °C, and the gradient program was set as follows: 0–11 min 4% B, 11–20 min linear increase 4–80% B, 20–33 min 80% B, 33–34 min linear decrease 80–4% B. Compounds were detected according to their secondary ions (MRM mode-Multiple Reaction Monitoring): lutein 568.1→569.4; β-carotene 536.4→537.3; α-tocopherol 430.4→165.1; γ-tocopherol 416.4→151.1; δ-tocopherol 402.4→137.1, with the same MRMs used for quantification (analyzed compounds produced only these MRMs in relatively high abundance). Source was ESI (ElectroSpray Ionisation) set to gas temperature 350 °C, gas flow 11 l/min, nebulizer 15 psi, capillary 4000 V in positive mode. Compounds were detected according to their secondary ions (MRM mode) and their concentrations were calculated according to 10-point calibration curves.

#### 2.2.2. Total Low Molecular Weight Antioxidant Activity (TAA)

TAA was measured using the DPPH method according to Brand-Williams et al. [51] with some modifications adapting the protocol to 96-well microtitre plates and to the measurement of absorbance with microtitre plate reader [52]. A solution of 0.5 mM of stable free radical 1,1-diphenyl-2-picrylhydrazyl (DPPH, SIGMA, Munich, Germany) in methanol was used. Absorbance was determined after 30 min of the reaction at 37 °C at 515 nm using reader Model 680 (Bio-Rad Laboratories, Hercules, CA, USA). Trolox was used as the standard in the following eight concentrations: 0.75, 1, 1.25, 1.5, 1.75, 2, 2.5, and 3 nM. Each concentration was pipetted (17.5 μL) into three wells and then 250 μL of 0.5 mM DPPH solution was added on each microtiter plate. Trolox equivalents for each measurement were calculated using the equation of linear regression from the calibration curve presenting linear relationships between absorbance and different Trolox concentrations. The results were expressed as μmoles of Trolox equivalents per 1 g of oil, macerate or oil cake.

For each type of oil cake, 200 µL samples of approx. 0.5 g were weighed. After adding 1.5 mL of 50% ethanol (Ethanol 96%, EMSURE, Reag. Ph Eur, Merck, Munich, Germany), the samples were shaken for 2 h at room temperature and in the dark on a Yellow Line OS 5 Basic shaker (560 cycles/min). Next the samples were centrifuged for 20 min. at 18,000× *g* (MPW-350R) and the supernatant was used for TAA measurements. Each sample was measured three times on separate plates by pipetting 10 μL of its supernatant and then 250 μL of 0.5 mM DPPH solution into 3 wells for each measurement.

For each oil and macerate, three 200 µL samples were pipetted into 1.5 mL vials and their weight was determined. Next, after adding 500 µL absolute ethanol (EMPLURA Ethanol absolute, Merck, Munich, Germany), the samples were shaken for 2 h at room temperature and in the dark on a Yellow Line OS 5 Basic shaker (560 cycles/min). Next, after phase separation, each sample was measured three times on separate plates by pipetting 50 µL from the upper alcoholic phase and then 250 μL of 0.5 mM DPPH solution into 3 wells of the microtiter plate.

#### 2.2.3. Total Dietary Fibre 

Dietary fibre content was determined with Total Dietary Fiber Assay Kit (Megazyme Ltd., Ireland) according to the procedure recommended by the producer. In brief, homogenized samples were washed with hexane to remove fat, then dried. Dry samples were mixed with MES-TRIS blend buffer solution (pH 8.2) and digested with α-amylase and later with protease. Then pH was changed to 4.1–4.8 and the samples were digested with amyloglucosidase. Finally, dietary fibre was precipitated with ethanol and collected in a crucible. After that crucibles with samples were dried and weighted. Additionally, the content of residue proteins (through the measurement of N content) and ash (through incineration at 525 °C) was determined in each sample and subtracted from the final result. Measurements were carried out in 6 replicates (3 per protein weight and 3 per ash weight) from the pooled sample for each combination plus 2 blanks for a total of 50 samples.

#### 2.2.4. Vitamin C

Ascorbic acid content was determined with Ascorbic Acid Assay Kit (Megazyme Ltd., Ireland) according to the procedure recommended by the producer. In short, homogenized samples were washed with hexane to remove fat and dried. Dry samples were mixed with metaphosphoric acid buffer, mixed, sonicated and centrifuged. Supernatant was collected and mixed with MTT (3-(4,5-dimethylthiazolyl-2)-2,5-diphenyltetrazolium bromide) solution. After 3 min PMS (5-methylphenazinium methosulphate) solution was added and finally absorbance (A) at 578 nm was read with Synergy 2 Microplate Reader (BioTek Instruments, Inc., United States) microplate reader. The ascorbic acid content (C) was calculated according to 10-point calibration curve according to the following equation:

A(sample) − A(blank) = 0.0025113 × C [ug/mL] + 0.0029987(1)

Measurements were carried out in 3 replicates from the pooled sample for each combination for a total of 24 samples.

### 2.3. Statistical Analysis

**Figure 1:**
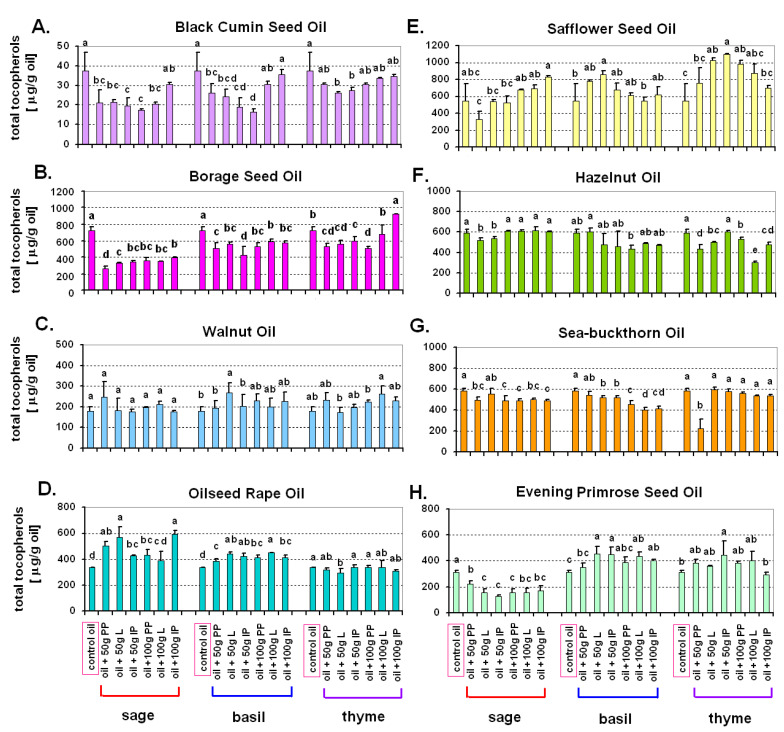
The impact of the addition of herbs (sage—*Salvia officinalis* L., basil—*Ocimum basilicum* L., thyme—*Thymus vulgaris* L.) on the content of total tocopherols in oils cold-pressed from the seeds of eight different species. (**A**) Black Cumin Seed Oil, (**B**) Borage Seed Oil, (**C**) Walnut Oil, (**D**) Oilseed Rape Oil, (**E**) Safflower Seed Oil, (**F**) Hazelnut Oil, (**G**) Sea-buckthorn Oil, (**H**) Evening Primrose Seed Oil. Control oil–cold-pressed oil without any additives. Values marked with the same letters are not significantly different according to the Duncan test (*p* ≤ 0.05); statistical analyses made separately for individual herbs. PP—aerial plant part cut into pieces; L—leaves only; IP—intact aerial plant part.

**Figure 2:**
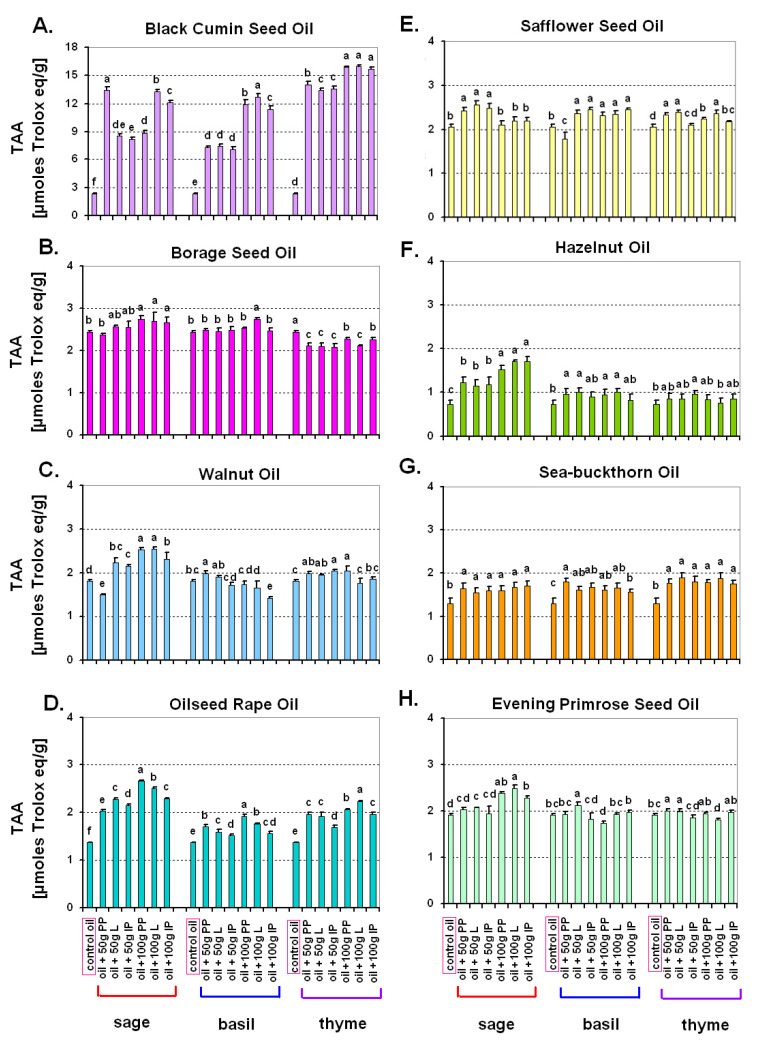
The impact of the addition of herbs (sage—*Salvia officinalis* L., basil—*Ocimum basilicum* L., thyme—*Thymus vulgaris* L.) on total low molecular weight antioxidant activity (TAA) of oils cold-pressed from the seeds of eight different species. (**A**) Black Cumin Seed Oil, (**B**) Borage Seed Oil, (**C**) Walnut Oil, (**D**) Oilseed Rape Oil, (**E**) Safflower Seed Oil, (**F**) Hazelnut Oil, (**G**) Sea-buckthorn Oil, (**H**) Evening Primrose Seed Oil. Control oil—cold-pressed oil without any additives. Values marked with the same letters are not significantly different according to a Duncan test (*p* ≤ 0.05); statistical analyses made separately for individual herbs. PP—aerial plant part cut into pieces; L—leaves only; IP—intact aerial plant part.

**Figure 3:**
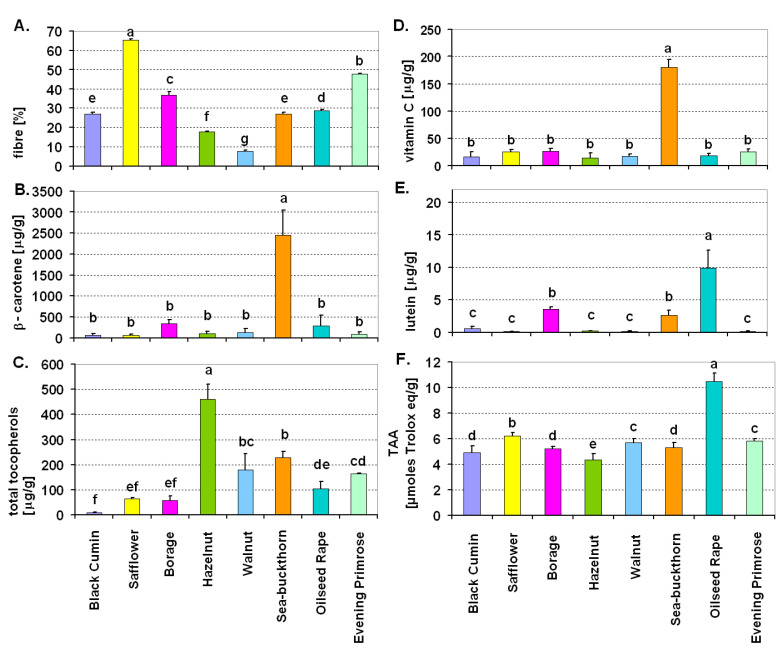
Contents of fibre (**A**), β-carotene (**B**), total tocopherols (**C**), vitamin C (**D**), lutein (**E**) and total low molecular weight antioxidant activity (TAA) (**F**) in oil cakes of eight species after cold-press of oil. Values marked with the same letters are not significantly different according to the Duncan test (*p* ≤ 0.05).

Statistical analyses (ANOVA, post-hoc test) were performed using Statistica 13 (StatSoft, Inc., Tulsa, OK, USA). The number of repetitions (*n*) for a particular measurement and information about what constitutes a repetition is provided in the description of a particular analytical method. In the case of tocopherol content (Figure 1, Figures S1, S2 and S3) and antioxidant activity (Figure 2) for a particular oil, the Duncan’s test (*p* ≤ 0.05) was used to compare mean values for each herb treatment (sage, basil, thyme) separately. In the case of chemical composition of oil cakes (Figure 3) [fibre, β-carotene, total tocopherols (including particular tocopherols—Figure S4), vitamin C, lutein] and antioxidant activity of oil cakes, mean values obtained for all eight tested oil cakes were compared using the Duncan’s test (*p* ≤ 0.05). Mean values in Figure 1, Figure 2 and Figure 3 and Figures S1–S4 are provided with standard deviation (±SD). Brief information about the statistical approach is included in the descriptions of all the figures.

## 3. Results

### 3.1. Total Tocopherols in Oils After the Addition of Herbs 

As expected, oils from different plant species were differentiated with regard to the content of total tocopherols. Total tocopherols were calculated as the sum of measured α-tocopherol, γ-tocopherol and δ-tocopherol (Figure 1A–H). The content of particular tocopherols in all eight tested oils was also different (data provided as Figures S1–S3, A-H in the Supplementary Material). The lowest content of total tocopherols was found in black cumin seed oil (less than 40 μg/g of control oil; Figure 1A). The highest amount of total tocopherols was noted in borage seed oil (about 800 μg/g of control oil; Figure 1B). In almost all control oils (without additives) the level of α-tocopherol was the highest (Figure S1). The exceptions were evening primrose seed oil, with the highest level of γ-tocopherol (Figure S2H) and borage seed oil characterized by the highest level of δ-tocopherol (Figure S3B). Compared to the dominant tocopherol, the remaining tocopherols were usually found in much lower quantities—even several hundred times lower. The effect of herbal supplementation on total tocopherols was very diverse and depended on the base oil and the species of the herb, ranging from a positive effect (an increase in the level of tocopherols) through no effect, to a negative effect (a decrease in the level of tocopherols).

In the case of black cumin seed oil (Figure 1A), the addition of sage and basil lowered the level of tocopherols compared to the control (oil without additives), except for the addition of 100 g of intact plants (IP) and in case of basil also 100 g of leaves (L). The addition of thyme did not affect tocopherols in black cumin seed oil, except for the addition of 50 g of leaves (L) and 50 g of intact plants (IP), which caused a decrease in total tocopherols by about 20% (Figure 1A). 

The addition of herbs lowered the level of total tocopherols in borage seed oil (Figure 1B). Higher levels of tocopherols than in the control oil were achieved only after the addition of thyme (100 g of intact aerial plant part (IP); Figure 1B). 

The addition of herbs to walnut oil did not change the level of total tocopherols (Figure 1C). The only exception was the addition of 50 g of basil leaves (statistically significant increase of tocopherols noted; Figure 1C). 

The addition of sage and basil (but not thyme) increased the level of total tocopherols in oilseed rape oil by, on average, 20% but sometimes even two-fold (in the case of 50 g of leaves (L) and 100 g of intact plants (IP); Figure 1D). 

In the case of safflower oil, the addition of sage and basil had no statistically significant effect on total tocopherols (Figure 1E). The only exception was the addition of 50 g of basil leaves (L), which caused a statistically significant increase in tocopherol level in this oil. The addition of thyme generally increased the level of tocopherols in safflower oil (Figure 1E), especially in the case of 50 g of leaves (L), 50 g of intact plant (IP) and 100 g of plants cut into pieces (PP). In other cases, the amount of total tocopherols was also elevated but not in a statistically significant way. 

The addition of sage and basil did not change or in some cases slightly decreased the content of tocopherols in hazelnut oil (from common hazel) (Figure 1F). The addition of thyme mostly reduced the content of tocopherols in this oil (Figure 1F).

The addition of sage and basil did not change or (especially in the amount of 100 g) decreased tocopherols in sea-buckthorn oil (Figure 1G). The addition of thyme generally did not change the content of tocopherols. Only after the addition of 50 g of thyme plants cut into pieces (PP), a significant decrease of tocopherols was noted (Figure 1G). 

The addition of sage decreased the amount of tocopherols in evening primrose seed oil (Figure 1H), in comparison to control oil without additives. The addition of basil generally increased the content of tocopherols. Thyme had no effect on the level of tocopherols, with the exception of 50 g of intact thyme plants (IP), which caused a statistically significant increase in tocopherol levels (Figure 1H).

### 3.2. Total Low Molecular Weight Antioxidant Activity (TAA) of Oils After Addition of Herbs 

As in the case of tocopherols, the addition of herbs caused a wide range of changes in total antioxidant activity of oils cold-pressed from the seeds of eight plant species (Figure 2A–H). A spectacular (manifold) increase of total antioxidant activity was achieved through the addition of each of the three herbs to black cumin seed oil (Figure 2A). Generally, the addition of 100 g of herbs was more effective. Unambiguously good effects were noted also after the addition of the three herbs to oilseed rape oil and sea-buckthorn oil (Figure 2D,G). In the case of safflower seed oil and hazelnut oil, total antioxidant activity was elevated, especially after the addition of sage and basil (Figure 2E,F). Relatively weak effects were noted after the addition of herbs to borage seed oil and evening primrose seed oil (Figure 2B,H).

### 3.3. Selected Chemical Components in Oil Cakes of Eight Plant Species After Cold-Press of Oil 

The oil cakes were characterized by different chemical composition, which, as expected, was influenced by the plant species from which they were obtained (Figure 3A–F, Figure S4A–C). The highest amounts of fibre were found in safflower (more than 60%) and evening primrose oil cakes (about 50%) (Figure 3A). In the case of other oil cakes, the amount of fibre was lower, with statistically significant differences between species. The highest amount of β-carotene (about 2500 μg/g) was recorded in sea-buckthorn oil cake (Figure 3B). The highest total tocopherol content was found in hazelnut oil cake (Figure 3C). In hazelnut oil cake the dominant tocopherol was α-tocopherol and in borage oil cake it was δ-tocopherol (similarly as in respective oils) (Figure S4A,C). In walnut oil cake, mainly γ-tocopherol remained after oil extraction (Figure S4B). The highest amount of vitamin C was found in sea-buckthorn oil cake, while in the other oil cakes, the amount of vitamin C was comparatively low (Figure 3D). Among the examined oil cakes, the highest lutein content was found in oilseed rape oil cake (Figure 3E). Particularly low content of lutein (below 0.2 μg/g, Figure 3E) was noted in oil cakes of safflower, walnut, hazelnut and evening primrose. Among the examined oil cakes, the highest total antioxidant activity was noted for oilseed rape oil cake (more than 10 μmoles of Trolox equivalents). In other oil cakes, antioxidant activity was comparable but lower by half than in oilseed rape (Figure 3F).

## 4. Discussion

Antioxidant properties are one of the most important criteria for quality assessment of food products. Generally, antioxidants include a large group of chemical compounds with different chemical structure—for example tocopherols but also phenols or carotenoids. Apart from vegetables, fruits, herbs and spices, these compounds can be found in plant oils, especially cold-pressed oils, which have high nutritional properties. 

This study shows that the effect of herbal supplementation on total tocopherol and total antioxidant activity of cold-pressed oils depended on both the base oil and the species of the added herb. The addition of herbs to the tested oils had different effects on their composition. An illustration of this is the case of evening primrose oil, in which the addition of sage lowered the level of tocopherols, the addition of basil increased the content of these compounds, and the addition of thyme had no effect. The presented research demonstrates that the composition of the macerate is affected by all three factors—the type of herb, its amount (50 g or 100 g), and the supplementation method (PP, L, IP). This is in agreement with other authors such as Tongnuanchan and Benjakul [53], according to whom the extraction method and part of the plant used for the extraction (roots, seeds, fruits, leaves, etc.) has a significant impact on the profile of bioactive compounds in natural extracts and essential oils of these plants. 

Tocopherols belong to the main components of the unsaponifiable fraction of vege-table oils, which are present in varying quantities. They are natural antioxidants occurring in four forms: α, β, γ and δ, which, show different antioxidant activity [54]. In the literature, many studies can be found proving that the content of individual tocopherols (or other bioactive compounds) is influenced by many factors, such as the plant variety from which the oil was pressed, as demonstrated by López Ortíz et al. [55], who showed differences in the content of α-tocopherol (14.8–21,8 mg/100 g of oil) between seven varieties of olives. The level and profile of tocopherols in oilseeds (e.g., sunflower, soybean or oilseed rape) is also highly dependent on environmental conditions, particularly temperature, and much less on the genotype [56]. In accordance with previously published data [57,58], this study also identified α- and γ-tocopherols as the main tocopherols in vegetable oils and fats. According to Schwartz et al. [54], Grilo et al. [59], and Shahidi and de Camargo [60] γ-tocopherol is the main form of tocochromanol present in cold-pressed oils. In most of the oils discussed in this work, α-tocopherol was the most abundant, followed by γ-tocopherol. The exception was borage seed oil, where the dominant tocopherol form was δ-tocopherol, and evening primrose oil, where it was γ-tocopherol, and these results are consistent with the studies of Fabrikov et al. [61], Hudson [62] and Christie [63]. As regards total tocopherols, some of the tested oils naturally contained relatively high content of tocopherols (even about 600–800 μg/g [hazelnut oil or borage seed oil]). In such cases, herbal supplementation was not very effective. An exception was safflower seed oil, with a similarly high content of native tocopherols, where the addition of thyme elevated total tocopherols even to the level higher than 1 mg/g. These combinations of oil with herbal raw material, where tocopherol content increased are most likely the result of tocopherol extraction from the herbal raw material. 

The biological activity of tocopherols, which results from their antioxidant properties and differs between their various forms, is crucially important from the nutritional point of view. It is the highest for α-tocopherol, lower for γ-tocopherol (10% α-tocopherol), and the lowest for δ-tocopherol (3% α-tocopherol) [64]. Taking into account these differences between tocopherols, safflower, hazelnut and rapeseed oils are the most valuable because they contain the highest amount of α-tocopherol. Safflower oil is particularly remarkable, as it not only contains the highest level of total tocopherols, but over 99% of it is the most active α-tocopherol. This high α-tocopherol content makes safflower oil an excellent dietary source of vitamin E, but it is also the reason for its low thermal stability in high temperature applications such as deep frying or machine lubrication [23,65]. In some of the tested combinations a decrease in tocopherol content was observed, possibly caused by the extraction of compounds reacting directly or indirectly with tocopherols. Such compounds may include chlorophyll, which in free form becomes a source of reactive oxygen species (ROS) [66]. Under conditions where hydrophobic oil is the solvent, the generated ROS will react primarily with hydrophobic antioxidants, including tocopherols. A more detailed comparison of the content of individual bioactive compounds (including tocopherols) in, among others, black cumin, safflower, hazelnut, walnut and oilseed rape oil can be found in the study of Ramadan et al. [67].

In addition to the level of tocopherols, total low molecular weight antioxidant activity (TAA) expressed as μmol of Trolox eq/g was also measured in the study. The addition of sage caused a significant increase in total antioxidant activity in every oil, whereas the other herbs did not cause such an obvious effect. This can be explained by the highest antioxidant activity of the compounds contained in sage in comparison to the other tested herbs. According to Hossain et al. [68], this activity is two-fold higher in sage than in basil or thyme. In our experiment, TAA also depended on the properties of the base oil. The highest values of TAA were obtained in the case of black cumin seed oil after the addition of each of the three herbs, but relatively high values were also recorded in oilseed rape oil.

Interestingly, however, a comparison of tocopherol content and TAA showed that there was no direct connection between them. It can be explained by the fact that the DPPH method of TAA measurement also quantifies other antioxidants, e.g., glutathione, ascorbic acid, phenols, polyphenols, flavonoids, anthocyanins, polysaccharides, and tannins [69], thus tocopherols were not the only players responsible for the antioxidant properties of the tested oils. The most striking example was the comparison of safflower oil—which was characterized by the highest tocopherol content—with black cumin seed oil. Black cumin seed oil contained several times less tocopherols than safflower oil, but simultaneously had the highest total antioxidant activity, especially after supplementation with herbal raw material. In this case other substances, such as aromatic compounds, must have been responsible for the antioxidant properties of this oil (probably also other oils with high TAA values as well) [70]. According to Ali and Blunden [71], black cumin seed oil contained particularly high quantities of aromatic compounds among the oils used in this study. This finding was partly confirmed also by the results of our preliminary research. Black cumin seed oil was characterized by extremely high content of essential oils in comparison to other oils (Table S1). According to Kowalski et al. [72], fat aromatization method affects the content of volatile compounds, whose concentration, in turn, does not necessarily enhance the antioxidant properties.

Apart from the characteristics of cold-pressed oils and macerates, the study also analyzed selected nutritional values of oil cake left after pressing the oil. According to literature, protein and dietary fibre are the dominant dry matter components of oil cake [48]. Our research also showed a high proportion of fibre (Figure 3A)—on average 30% up to maximum about 70% (in safflower oil cake). The tested oil cakes were also characterized by high antioxidant activity. In the oils, TAA values were usually around 2 μmol Trolox eq/g, while in the oil cakes they ranged from over 4 to even 10 μmol Trolox eq/g. One of the causes could be the fact that a characteristic feature of each oil cake type is phenolic compound content [73]. Most of the phenolic compounds and other fat-insoluble antioxidants do not pass into the oil but remain in the oil cake, influencing antioxidant activity [21,47,74,75,76]. These authors showed high content of phenolic compounds and high antioxidant activity in extracts from evening primrose seed oil cake (tested also in our study). Shahidi et al. [74] identified isoflavones, proanthocyanidins and gallic acid derivatives in the above-mentioned oil cake. Our study additionally showed that oil cake also retains relatively large quantities of other antioxidants such as tocopherols (hazelnut), β-carotene and vitamin C (sea buckthorn).

To summarize, the effect of herbal supplementation on total tocopherols and total antioxidant activity of macerates depended both on the base oil and the species of the added herb. For tocopherols, some of the tested oils naturally contained relatively high content of tocopherols—even about 600–800 μg/g [hazelnut oil and borage seed oil]. In such cases, herbal supplementation was not very effective. One exception was safflower seed oil, with a similarly high amount of native tocopherols, where the addition of thyme increased total tocopherols even to the content of about 1 mg/g. As for total antioxidant activity, record values were noted in the case of black cumin seed oil after the addition of each of the three herbs, but relatively high values were also noted in oilseed rape oil.

The presented research demonstrates that the antioxidant properties of the tested oils are influenced by many factors. Various bioactive compounds from herbal raw material or oils may positively or negatively affect the antioxidant properties of the macerates. The results showed that tocopherol content was not the only factor determining total antioxidant activity, and that essential oils or another biological compounds were at least as important.

The oil cakes were characterized by different chemical composition, which was obviously influenced by the plant species from which they were obtained. The greatest amounts of β-carotene and vitamin C were found in sea-buckthorn oil cakes. The highest amount of tocopherols was found in hazelnut oil cakes.

## 5. Conclusions

Total tocopherols and total antioxidant activity of macerates depended both on the species from which the oil was cold-pressed and the species of the added herb. This study can serve as a starting point for the development of healthy oils and oil cakes with high antioxidant contents, but simultaneously one should bear in mind that the addition of herbs to the tested oils had different effects on their composition (from positive influence on antioxidant properties, to neutral or even negative effects). On the other hand, one must also remember that the results are based on two methods of analysis (TAA, tocopherols). In the future further analysis, allowing more detailed characterization of the material, would be interesting. Generally, the addition of herbal preparations to the oils could be considered a sophistication which can alter the original properties of the oils.

Taking into consideration the results of total tocopherol content and total antioxidant activity as well as the popularity of oilseed rape oil in the cuisine of many countries (in comparison to other tested oils), it would be advisable to add sage and basil herbs to oilseed rape oil to enrich it with greater antioxidant properties for the purpose of healthy nutrition.

Oil cakes are still rich in substances of nutritional value and thus can be further used for the preparation of food additives. Our research could provide the incentive to maximize efficient use of plant raw materials, particularly less common species, and increase the health benefits of products containing them.

## Figures and Tables

**Table 1:** Type and characteristics of the plant materials used in the experiment.

Type of Material	Characteristics
Raw Material	Seeds: black cumin (*Nigella sativa* L.), borage (*Borago officinalis* L.), evening primrose (*Oenothera biennis* L.), safflower (*Carthamus tinctorius* L), walnut (*Juglans regia* L)., common hazel (*Corylus avellana* L.), and oilseed rape (*Brassica napus* L.) Berries: sea-buckthorn (*Hippophae rhamnoides* L.) (first macerated with rapeseed oil in a 1:1 proportion)
Base Oils (Control Oils)	8 base oils cold-pressed form seeds and berries at slow-speed screw press (temperature range of 33–35 °C)
Oil Cakes	8 oil cakes-residue from the oil pressing process
Herbal Material (Herbs) Used for Preparation of Macerates	3 herbs: sage (*Salvia officinalis* L.), common thyme (*Thymus vulgaris* L.), basil (*Ocimum basilicum* L.) Forms and weights of herbal material: whole aerial part of the plant (intact plant, IP)—50 g and 100 g aerial part of the plant cut into 1 cm sections (plant pieces, PP)—50 g and 100 g whole leaves (L)—50 g and 100 g.
Macerates (Base Oil + Herb)	144 macerates, obtained by 10-day maceration at 15 °C 144 combinations = 8 base oils × 3 herbs (sage, thyme, basil) × 3 forms (IP, PP, L) × 2 weights (50 g and 100 g)

## Data Availability

All data is contained within the article. The datasets used and analyzed during the current study are available from the corresponding author on reasonable request.

## Supplementary Materials

The following are available online at https://www.mdpi.com/article/10.3390/antiox10050781/s1, Figure S1: The impact of the addition of herbs (sage—*Salvia officinalis* L., basil—*Ocimum basilicum* L., thyme—*Thymus vulgaris* L.) on α-tocopherol content in oils cold-pressed from seeds of eight different species. Figure S2: The impact of the addition of herbs (sage—*Salvia officinalis* L., basil—*Ocimum basilicum* L., thyme—*Thymus vulgaris* L.) on γ-tocopherol content in oils cold-pressed from seeds of eight different species. Figure S3: The impact of the addition of herbs ((sage—*Salvia officinalis* L., basil—*Ocimum basilicum* L., thyme—*Thymus vulgaris* L.) on δ-tocopherol in oils cold-pressed from seeds of eight different species. Figure S4: Contents of α-tocopherol (A), γ-tocopherol (B) and δ-tocopherol (C) in oil cakes of eight species after cold-press of oil. Table S1: The impact of addition of herbs on total content (sum) of essential oils [μg/g] in oils cold-pressed from eight plant species–preliminary analysis [77].
