# Supplementary material for: Herbal Additives Substantially Modify Antioxidant Properties and Tocopherol Content of Cold-Pressed Oils
Source: Antioxidants (Basel). 2021 May 14;10(5):781. doi: 10.3390/antiox10050781 (PMC8157206; doi:10.3390/antiox10050781)
Supplement: Supplementary file 1 [file antioxidants-10-00781-s001.zip › antioxidants-1212309-supplementary.pdf]

Supplementary Materials:

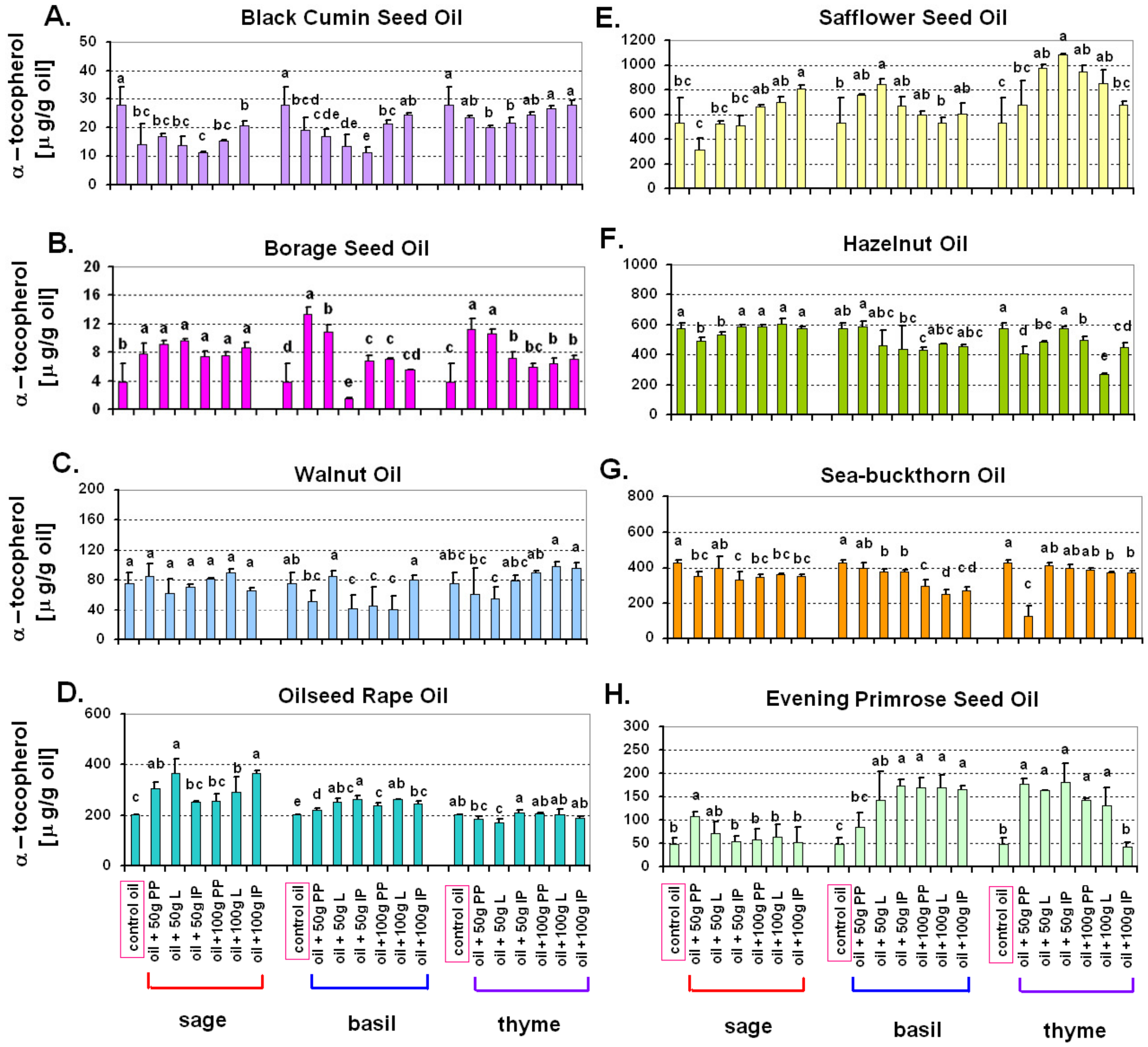

Figure S1: The impact of the addition of herbs (sage—*Salvia officinalis* L., basil—*Ocimum basilicum* L., thyme—*Thymus vulgaris* L.) on α-tocopherol content in oils cold-pressed from seeds of eight different species. Control oil – cold-pressed oil without any additives. Values marked with the same letters are not significantly different according to the Duncan test (*p* 0.05); statistical analyses made separately for individual herbs. PP – aerial plant part cut into pieces; L—leaves only; IP—intact aerial plant part.

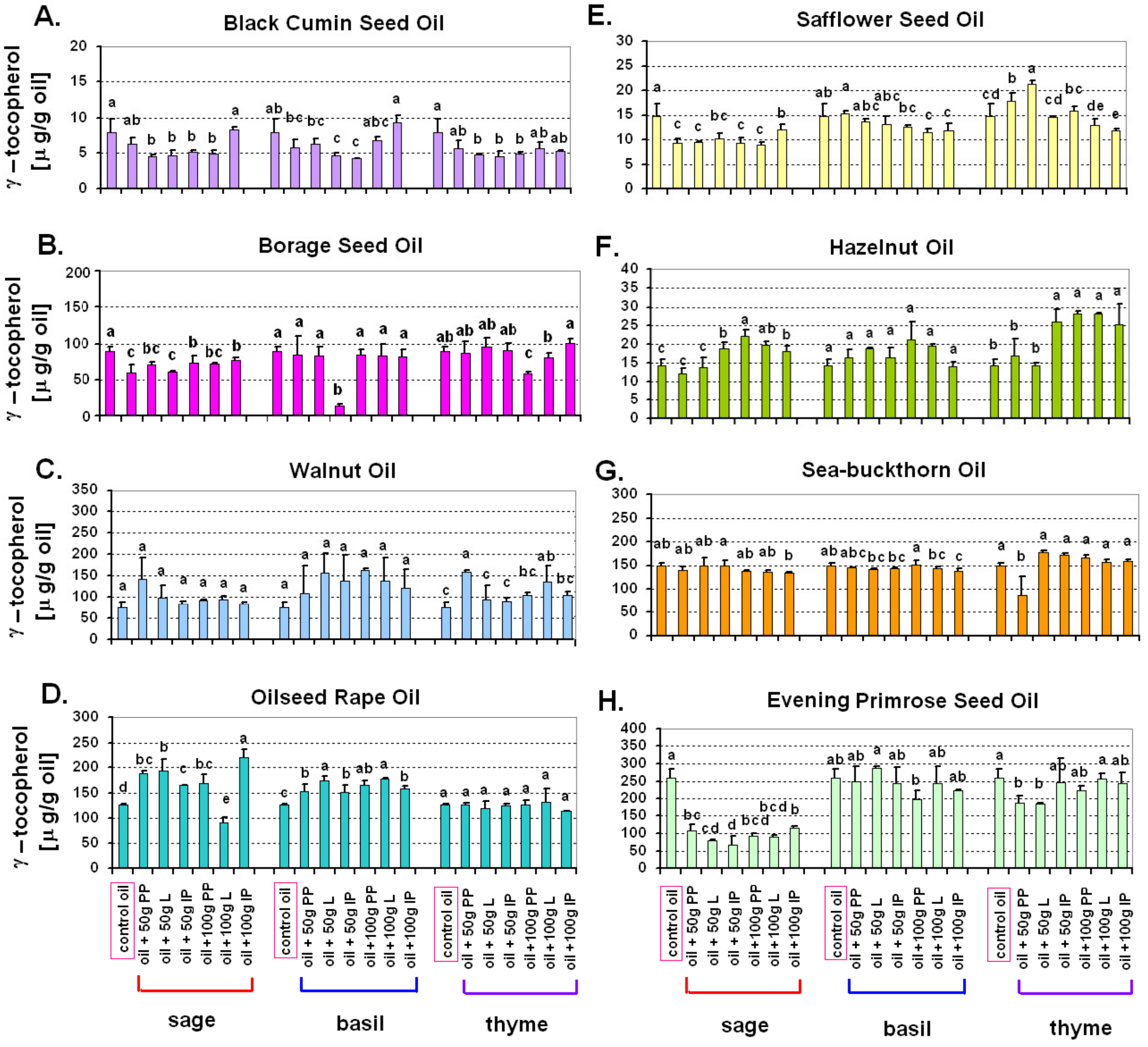

Figure S2: The impact of the addition of herbs (sage—*Salvia officinalis* L., basil—*Ocimum basilicum* L., thyme—*Thymus vulgaris* L.) on γ-tocopherol content in oils cold-pressed from seeds of eight different species. Control oil – cold-pressed oil without any additives. Values marked with the same letters are not significantly different according to the Duncan test (*p* 0.05); statistical analyses made separately for individual herbs. PP—aerial plant part cut into pieces; L—leaves only; IP—intact aerial plant part.

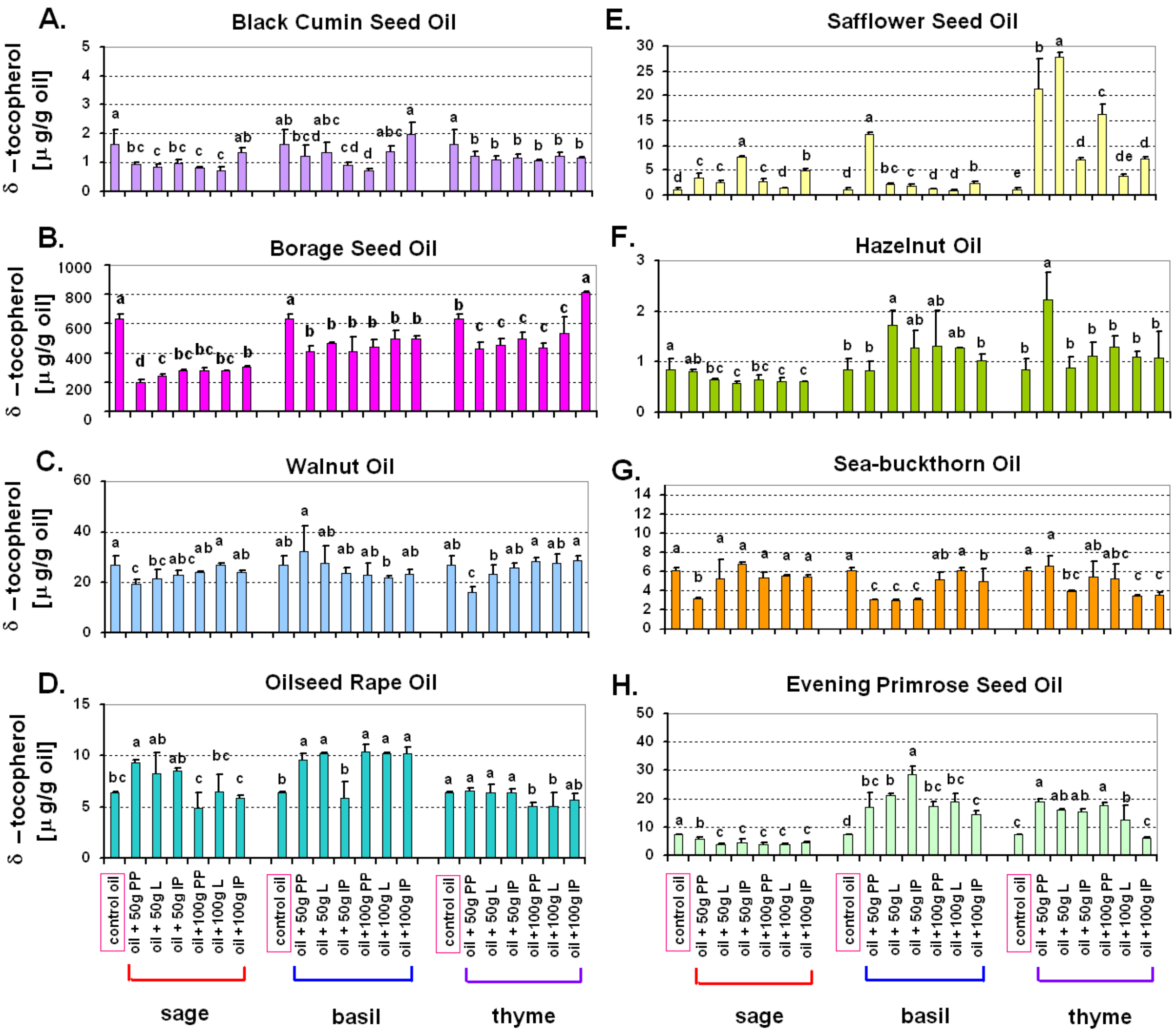

Figure S3: The impact of the addition of herbs (sage—*Salvia officinalis* L., basil—*Ocimum basilicum* L., thyme—*Thymus vulgaris* L.) on δ-tocopherol in oils cold-pressed from seeds of eight different species. Control oil – cold-pressed oil without any additives. Values marked with the same letters are not significantly different according to the Duncan test (*p* 0.05); statistical analyses made separately for individual herbs. PP—aerial plant part cut into pieces; L—leaves only; IP—intact aerial plant part.

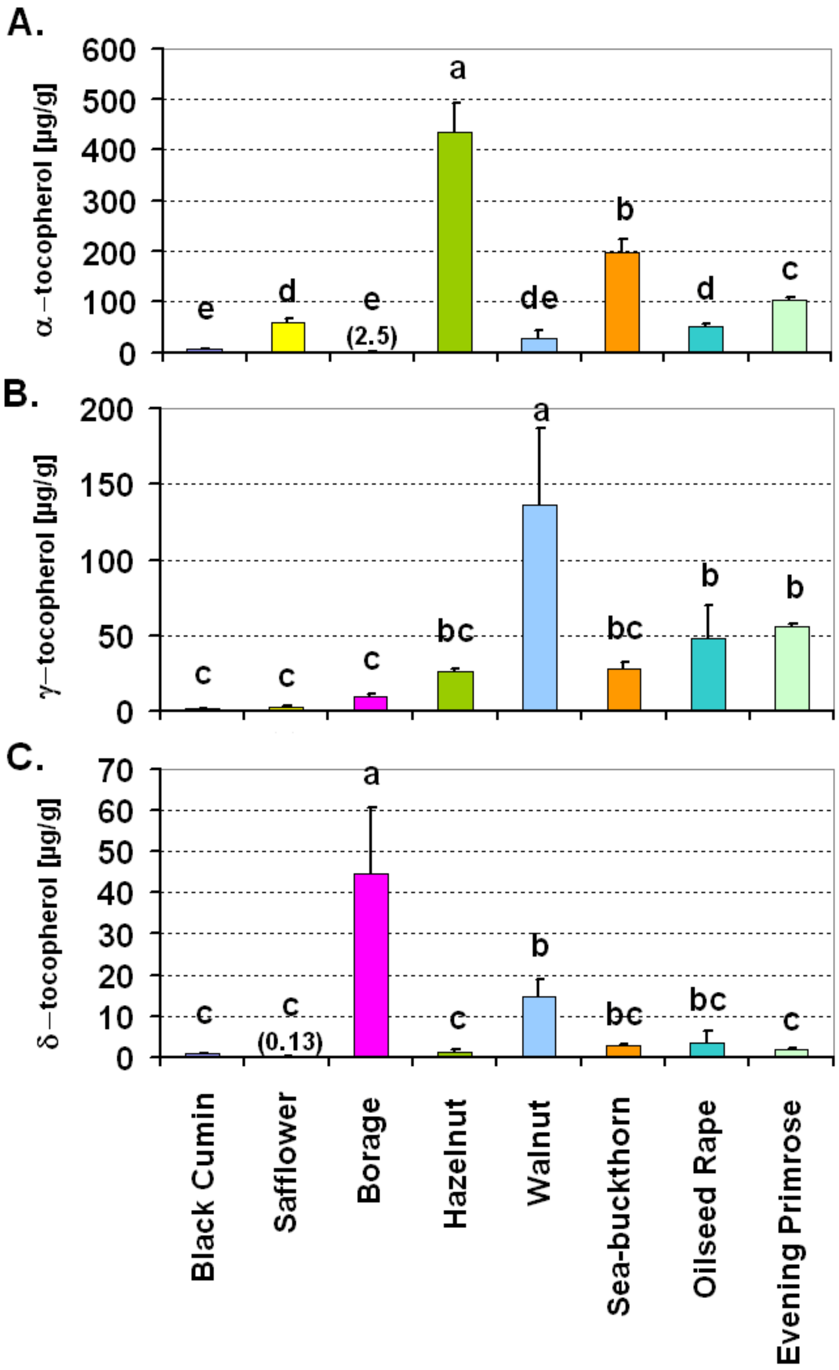

Figure S4: Contents of α-tocopherol (**A**), γ-tocopherol (**B**) and δ-tocopherol (**C**) in oil cakes of eight species after cold-press of oil. Values marked with the same letters are not significantly different according to the Duncan test (*p* 0.05). Extremely low values are provided in parenthesis.

Table 1. The impact of addition of herbs on total content (sum) of essential oils [μg/g] in oils cold-pressed from eight plant species–preliminary analysis.

Oil + added herb	Black Cumin Seed Oil	Safflower Seed Oil	Borage Seed Oil	Hazlenut Oil	Walnut Oil	See-Buckthorn Seed Oil	Oilseed Rape Oil	Evening Primrose Seed Oil
Oil	16920	9	3	4	65	12	12	9
Oil + *Salvia officinalis* L. 50 PP	15444	159	46	127	155	79	93	50
Oil + *Salvia officinalis* L. 50 L	17359	244	72	120	155	84	200	80
Oil + *Salvia officinalis* L. 50 IP	14944	197	73	96	114	77	123	57
Oil + *Salvia officinalis* L. 100 PP	17192	75	175	188	290	120	306	175
Oil + *Salvia officinalis* L. 100 L	12836	89	162	263	262	128	307	216
Oil + *Salvia officinalis* L. 100 IP	12926	75	205	241	198	127	280	138
Oil	16920	9	3	4	65	12	12	9
Oil + *Ocimum basillicum L.* 50 PP	20985	113	125	68	233	124	139	169
Oil + *Ocimum basillicum L.* 50 L	21401	191	169	77	246	110	177	208
Oil + *Ocimum basillicum L.* 50 IP	21128	158	131	52	121	120	134	153
Oil + *Ocimum basillicum L.* 100 PP	19119	190	231	118	316	175	313	260
Oil + *Ocimum basillicum L.* 100 L	17987	325	344	115	332	197	294	330
Oil + *Ocimum basillicum L.* 100 IP	18368	271	280	103	328	159	198	220
Oil	16920	9	3	4	65	12	12	9
Oil + *Thymus vulgaris L.* 50 PP	16225	210	289	76	65	155	61	219
Oil + *Thymus vulgaris L.* 50 L	16198	210	212	68	59	148	158	190
Oil + *Thymus vulgaris L.* 50 IP	15923	251	180	75	62	154	108	252
Oil + *Thymus vulgaris L.* 100 PP	12394	394	533	273	83	52	248	389
Oil + *Thymus vulgaris L.* 100 L	12585	396	378	170	93	55	203	387
Oil + *Thymus vulgaris L.* 100 IP	13664	480	292	165	80	51	176	410

Essential oils measured by modified method described by modified method of Silva-Flores et al. [77]. Control oil – cold-pressed oil without any additives. PP—aerial plant part cut into pieces, L—leaves only; IP—intact aerial plant part, 50–50 g of plant material, 100–100 g of plant material.
